# RNA-Cholesterol Nanoparticles Function as Potent Immune Activators *via* TLR7 and TLR8

**DOI:** 10.3389/fimmu.2021.658895

**Published:** 2022-01-21

**Authors:** Hannah-Lena Obermann, Ines I. Lederbogen, Jenny Steele, Jens Dorna, Leif Erik Sander, Konrad Engelhardt, Udo Bakowsky, Andreas Kaufmann, Stefan Bauer

**Affiliations:** ^1^ Institute for Immunology, Philipps-University Marburg, Marburg, Germany; ^2^ Department of Infectious Diseases and Pulmonary Medicine, Charité-Universitätsmedizin Berlin, Corporate Member of Freie Universität Berlin, Humboldt-Universität zu Berlin, and Berlin Institute of Health, Berlin, Germany; ^3^ Department of Pharmaceutics and Biopharmaceutics, Philipps-University Marburg, Marburg, Germany

**Keywords:** TLR7/8 ligand, RNA-cholesterol, nanoparticle, immunostimulation, dsRNA

## Abstract

The innate immune system senses viral and bacterial ribonucleic acid (RNA) *via* pattern recognition receptors (PRR) leading to subsequent activation of the immune system. One group of RNA sensors is formed by endosomal/lysosomal Toll-like receptors (TLR) such as TLR7 and TLR8. During viral or bacterial infection, immunostimulatory RNA is part of the pathogen reaching the endosomal/lysosomal compartment after cellular uptake. Synthetic single-stranded or double-stranded oligoribonucleotides (ORN) can mimic RNA from pathogens and are widely used as activating ligands for TLR7 and TLR8. However, one limitation in the use of synthetic ORN driven immune stimulation is the need for transfection reagents for RNA delivery into cells. Here we demonstrate that the conjugation of cholesterol to a double-stranded version of immunostimulatory RNA40 strongly enhanced RNA uptake into monocytes and plasmacytoid dendritic cells when compared to naked RNA. Cholesterol-conjugated RNA (RNA-chol) formed nanoparticles that were superior to RNA-liposomes complexes in regard to induction of type I interferon from human and murine plasmacytoid dendritic cells as well as proinflammatory cytokine production (e.g. TNF-α, IL12p70 or IL-6) in human monocytes. Furthermore, the RNA40-chol induced cytokines in human monocyte cultures supported T_H1_ and T_FH_ cell differentiation underscoring a strong adjuvant function of RNA-chol nanoparticles for adaptive immune responses. In summary, cholesterol-conjugated immunostimulatory RNA forms nanoparticles and functions as a potent immune adjuvant in human and murine immune cells. It further simplifies the use of immunostimulatory RNA by avoiding the need for liposomal transfection reagents.

## Introduction

The synthetic immunostimulatory toll-like receptor 7- (TLR7) and TLR8-ligand (TLR8L) ribonucleic acid 40 (RNA40), which is a single-stranded (ss) GU-rich sequence from the U5 region of HIV-1 RNA, and its potential to act as adjuvant or for immunotherapy has already been described (1). In addition, double-stranded (ds) short interfering (si) RNA is a potent inducer of interferon-α (IFN-α) secretion in murine plasmacytoid dendritic cells (pDC) through activation of TLR7 (2). TLR7 and TLR8 belong to a subgroup of pattern recognition receptors (PRR) that is located within the endosomal/lysosomal compartment (3) of immune cells and sense RNA degradation products: TLR7 recognizes guanosine and uridine moieties in ssRNA (4) and TLR8 detects uridine and a short oligonucleotide created by RNase T2 and/or RNase 2 (5–7). Since unmodified nucleic acids hardly interact with cell membranes and are highly prone to degradation by DNases or RNases, cellular uptake of synthetic TLRL has to be facilitated by complexing the nucleic acids to transfection reagents like DOTAP. A major drawback of this method is the additional step of complex formation, which has to be performed prior to adding the TLRL to target cells. For this reason, developing TLRL that can be directly added to and activate target cells is highly desirable. Among other molecules, cholesterol-conjugation has been shown to: i) improve nuclease resistance of oligodeoxynucleotides (ODN) (8), ii) mediate cellular uptake of siRNA across plasma membranes (9, 10) and iii) induce an immune response by ssRNA with phosphorothioate linkage (11). In addition, cholesterol-conjugation of non-methylated CpG-DNA leads to formation of nanoparticles by self-assembly (12). Conjugation of cholesterol to siRNA and antisense oligodeoxynucleotides and their cellular uptake as well as *in vivo* distribution has been extensively investigated (9, 10, 13–16). In contrast, cholesterol-conjugation to immunostimulatory phosphodiester RNA (e.g. dsRNA40) in order to activate the innate immune system has not been studied in detail. Due to the ability of macrophages and dendritic cells to incorporate nano- and microparticles *via* phagocytosis or endocytosis, these particles possess the potential to act as drug or adjuvant delivery system. In this study, we investigated the potential of a cholesterol-conjugated dsRNA [based on the sequence of the TLR7/TLR8 ligand RNA40 (1)] to form nano- or microparticles and activate TLR7 and TLR8. Therefore, we analyzed cellular uptake and the immunostimulatory potential of cholesterol-conjugated dsRNA40 by murine Flt3L-DC, human pDC and human monocytes and analyzed the contribution of RN40-chol-induced immunostimulation to T cell differentiation.

## Materials and Methods

### Ethics Statement

The local ethics committees of Justus-Liebig-University Giessen and Philipps-University Marburg approved the use of human blood samples for this study. For experiments with murine immune cells, mice were sacrificed, and tissue/organs removed. These experiments were performed in accordance with the National German welfare law §4 (3) TierSchG and §2 and Annex 2 (TierSchVerV) of the National Order for the use of animals in research and do not need the approval by a local ethics committee. According to the regulations, the number of mice used was reported to the animal welfare officer of the Philipps-University Marburg.

### Reagents

ssRNA40sense (5’-GCCCGUCUGUUGUGUGACUC), GU-rich sequence from the U5 region of HIV-1 RNA, nt 108-127 (1); ssRNA40-chol (5’-GCCCGUCUGUUGUGUGACUC-cholesteryl), ssRNA40antisense (5’-GAGUCACACAACAGACGGGC), ssRNA40anti-2’O-methyl (5’-GaGuCaCaCaAcAgAcGgGc) where small letters indicate 2’O-methylated positions and ssRNA40antisense-Alexa488 (ssRNA40antisense (5’-GAGUCACACAACAGACGGGC-Alexa488) were synthesized by IBA (Göttingen, Germany) or BioSpring GmbH (Frankfurt, Germany).The sequence of CpG-ODN 2216 (5’-GsGsGGGACGATCGTCsGsGsGsGsGsG), where ‘s’ depicts a phosphorothioate linkage, was synthesized by TIB MOLBIOL (Berlin, Germany). Resiquimod (R848), TL8-506 were purchased from InvivoGen (San Diego, USA), LPS from Sigma Aldrich (Steinheim, Germany) and DOTAP was purchased from Carl Roth (Karlsruhe, Germany) or Roche (Basel, Switzerland). Flt3-ligand (Flt3L) was prepared from an Flt3L-secreting cell line (H. Hochrein, Bavarian Nordic GmbH, Martinsried, Germany). Antibodies were purchased from BD Biosciences (rat anti-mouse B220-FITC), eBioscience (rat anti-mouse CD11c-APC), Miltenyi Biotec (mouse anti-human BDCA-2-FITC, mouse anti-human CD14-PE) and Biolegend (mouse anti-human CD3, mouse anti-human CD28, mouse anti-human CD4 and mouse anti-human IFN-γ).

### Annealing of dsRNA

For the generation of dsRNA40, ssRNA40sense and ssRNA40antisense were annealed. dsRNA40-chol was created by annealing ssRNA40-chol and ssRNA40antisense, whereas dsRNA40-2’OM was generated by annealing ssRNA40sense and ssRNA40anti-2’O-methyl. In brief, equal molar amounts of complementary single-stranded RNA molecules were combined in annealing buffer (20 mM HEPES, 150 mM NaCl) and the solution was heated to 95°C for ten minutes and slowly cooled down to room temperature. Formation of double-stranded RNA (final concentration of 20 µM) was verified by 15% non-denaturing polyacrylamide gel-electrophoresis.

### Mice

TLR7-deficient mice were established as described and have been backcrossed to C57BL/6 mice for at least 12 generations (17). C57BL/6 wild type mice were bred in the animal facility of the Philipps-University Marburg. Mice were kept under specific pathogen-free conditions in the animal facility of the Philipps-University Marburg at the biomedical research center.

### Cells

To differentiate Flt3L-induced mixed cultures of murine plasmacytoid and myeloid dendritic cells, bone marrow cells were seeded at 1.5x10^6^ cells/ml in Opti-MEM (Thermo Fisher Scientific, Waltham, USA) supplemented with 0.05 mM β-mercaptoethanol (Thermo Fisher Scientific, Waltham, USA), 100 U/ml penicillin (PAA, Cölbe, Germany), 100 µg/ml streptomycin (PAA, Cölbe, Germany), 1% FCS (Biochrom AG, Berlin, Germany) and cultured with the Flt3L containing supernatant in a 1:250 dilution for seven days. Bone marrow derived macrophages (BMDM) were generated using 20 ng/ml murine M-CSF (Peprotech, Rocky Hill, USA) with 5x10^6^ cells/10 ml in RPMI1640 supplemented with 0.05 mM β-mercaptoethanol (Thermo Fisher Scientific, Waltham, USA), 100 U/ml penicillin (PAA, Cölbe, Germany), 100 µg/ml streptomycin (PAA, Cölbe, Germany) and cultured for 5 days. Three days after isolation additional 20 ng/ml M-CSF was added. Human PBMC were isolated by Ficoll density gradient centrifugation using lymphocyte separation medium LSM1077 (PAA, Cölbe, Germany) according to the manufacturer’s recommendation. Untouched human pDC and untouched human monocytes were isolated with the plasmacytoid dendritic cell isolation kit II or the monocyte isolation kit II (Miltenyi Biotec, Bergisch-Gladbach, Germany) from PBMC according to the manufacturer’s recommendation. Cells were routinely analyzed by flow cytometry (FACSCalibur, BD Biosciences, San Jose, USA) to assess purity.

### Cell Stimulation

Murine Flt3L-induced DC and human monocytes were seeded at 2x10^5^ cells/well and human pDC were seeded at 0.15x10^5^ cells/well. Murine Flt3L-induced DC were cultured in Opti-MEM (Thermo Fisher Scientific, Waltham, USA) supplemented with 0.05 mM β-mercaptoethanol (Thermo Fisher Scientific, Waltham, USA), 100 U/ml penicillin (PAA, Cölbe, Germany), 100 µg/ml streptomycin (PAA, Cölbe, Germany), 1% FCS (Biochrom AG, Berlin, Germany). Human monocytes and pDC were cultured in RPMI 1640 (PAA, Cölbe, Germany) supplemented with 2 mM L-glutamine (PAA, Cölbe, Germany), 100 U/ml penicillin (PAA, Cölbe, Germany), 100 µg/ml streptomycin (PAA, Cölbe, Germany), 1x non-essential amino acids (PAA, Cölbe, Germany), 1 mM sodium pyruvate solution (PAA, Cölbe, Germany). Human pDC and monocytes were stimulated without serum.

Cells were stimulated with ssRNA40 or dsRNA40 complexed to DOTAP as well as dsRNA40-chol, and dsRNA40-chol-2’O-methyl at concentrations as indicated. After 20 hours supernatants were harvested and analyzed for cytokine secretion. Stimuli diluted in Opti-MEM in a total of 25 µl were mixed with 25 µl DOTAP-solution (2.5 µl DOTAP and 22.5 µl Opti-MEM) and incubated for ten minutes at room temperature before an equal volume of medium was added. For stimulation without complexing stimuli to DOTAP, DOTAP-solution was replaced by Opti-MEM. Cells stimulated with medium, DOTAP, CpG2216 (1 µM), LPS (1 µg/ml) in combination with R848 (2.5 µg/ml) served as negative and positive controls, respectively. Cells within 100 µl volume were incubated with 100 µl of stimuli in 96-well microplates (Greiner Bio-one, Gremsmünster, Austria). Cytokines were analyzed by ELISA with reagents from PBL (Piscataway, USA) for murine IFN-α or Peprotech (Rocky Hill, USA) for murine TNF-α, murine CXCL10, murine and human IFN-β or R&D Biosystems (Minneapolis, USA) for murine IL-6 and human IL-1β or BD Biosciences (San Jose, USA) for human IL-6 and TNF-α or Bender MedSystems (Wien, Austria) and BD Biosciences (San Jose, USA) for human IFN-α and eBioscience (San Diego, USA) for human IL-12p70.

### *In Vitro* T Cell Priming

Human PBMC were isolated as described above. CD4^+^ T cells were isolated from PBMC with the MagniSort Human CD4 T cell enrichment kit (eBioscience, San Diego, USA) according to the manufacturer’s recommendation. CD4^+^ T cells were seeded at 0.5x10^5^ cells/well within 100 µl RPMI 1640 (GIBCO/Invitrogen, Carlsbad, CA, USA) supplemented with 1% non-essential amino acids (Sigma, München, Germany), 1% HEPES (Sigma, München, Germany), 1% GlutaMAX supplement (Thermo Fisher Scientific, Waltham, USA), 1% penicillin/streptomycin (Sigma, München, Germany) and 10% heat-inactivated autologous plasma) into anti-CD3 (4 µg/ml) coated Corning^®^ 96-well plates (Corning, New York, USA). 100 µl of conditioned monocyte supernatant (monocytes were stimulated as described above) and 1 µg/ml anti-CD28 were added to the CD4^+^ T cells. After five days of incubation, cells were analyzed for intracellular IFN-γ, IL-21 and Bcl-6. Therefore, PMA (Sigma, München, Germany) and ionomycin (Sigma, München, Germany) were added at 50 ng/ml and 1 µg/ml to CD4^+^ T cells. Cells were incubated for 2.5 hours before 1x brefeldin A (eBioscience, San Diego, USA) and 1x monensin (Biolegend, San Diego, USA) were added and cells were incubated for additional 2.5 hours. Cells were harvested and stained for CD4 and intracellular IFN-γ, IL-21 and Bcl-6. For the staining of IFN-γ and IL-21, cells were fixed and permeabilized with cytofix/cytoperm fixation solution and perm/wash buffer as described in the operation manual of the manufacturer BD Biosciences. For the staining of Bcl-6, cells were fixed and permeabilized with the Foxp3/transcription factor staining buffer set (eBioscience, San Diego, USA) as described in the operation manual. Cells were analyzed with a FACS CANTO II and the FACS Diva software, which was available in the flow cytometry lab of the BCRT, Charité Universitätsklinikum Berlin.

### Photon Correlation Spectroscopy (PCS) and Laser Doppler Velocimetry (LDA)

The hydrodynamic diameter and zeta potential of the dsRNA-chol particles were measured by PCS and LDV respectively using Zetasizer Nano ZS (Malvern Instruments, Herrenberg, Germany) equipped with 10 mW HeNe laser at a wavelength of 633 nm at 25°C. A viscosity of 0.88 mPa×s and a refractive index of 1.33 of water at 25°C were assumed for data interpretation. Laser attenuation and measurement position were adjusted automatically by the instrument. The zeta potential was measured *via* electrophoretic mobility with laser Doppler velocimetry (LDA). The average values of the size intensity peak, the size volume peak, z-average (z-AvE) and zeta potential were calculated with data of three independent experiments ± standard deviation. Each sample was measured three times with at least 10 sub runs.

### Atomic Force Microscopy (AFM) Measurements

AFM was performed using a NanoWizard^®^ 3 atomic force microscope (JPK Instruments, Berlin, Germany). Silicon cantilevers (HQ : NSC14/AL_BS, MikroMasch Europe, Wetzlar, Germany) were used to measure the dsRNA40-chol particles. Measurements were performed in intermittent contact mode to avoid damage of the sample (18). Images were obtained by displaying the amplitude signal of the cantilever in the trace direction and the measured height mode in retrace direction. For diameter measurements the height mode signal in trace direction are used. The images of the particles were presented in amplitude mode.

### Confocal Laser Scanning Microscopy

Human pDC were incubated with 0.5 µM of Alexa488-labeled dsRNA40-chol, dsRNA40 or dsRNA40 complexed to DOTAP. Mixtures were prepared as described for cell stimulations. After 1.5 hours of incubation, cells were extensively washed to remove any free nucleic acids. Cells were then fixed with 2% paraformaldehyde (Carl Roth, Karlsruhe, Germany). Nuclei were stained with 0.1 µg/ml of DAPI (Merck, Darmstadt, Germany) and pictures were taken with a TCS SP5 confocal laserscanning microscope (original magnification x63) (Leica Microsystems, Wetzlar, Germany). Images were processed and plot profiles were generated with Fiji (ImageJ) software.

### Flow Cytometry

Primary cells were incubated with 0.5 µM of Alexa488-labeled dsRNA40-chol, dsRNA40 or dsRNA40 complexed to DOTAP. Mixtures were prepared as described for cell stimulations and confocal laser scanning microscopy. Cells were analyzed with a FACSCalibur (BD Biosciences, San Jose, USA).

### Statistical Analysis

Statistical significance was analyzed using unpaired t-test or one-way ANOVA followed by *post-hoc* tests as indicated to correct for multiple comparisons. Data calculations were performed using Graph Pad Prism 8.4.1 software. Bars show mean + SEM of biological replicates. The values of p < 0.05 were considered to be statistically significant (*p < 0.05, **p < 0.01, ***p < 0.001, ****p < 0.0001).

## Results

### Cholesterol-Conjugated dsRNA40 Induces Potent Immunostimulation in Plasmacytoid Dendritic Cells and Human Monocytes in a TLR7- and TLR8-Dependent Manner

**Figure 1:**
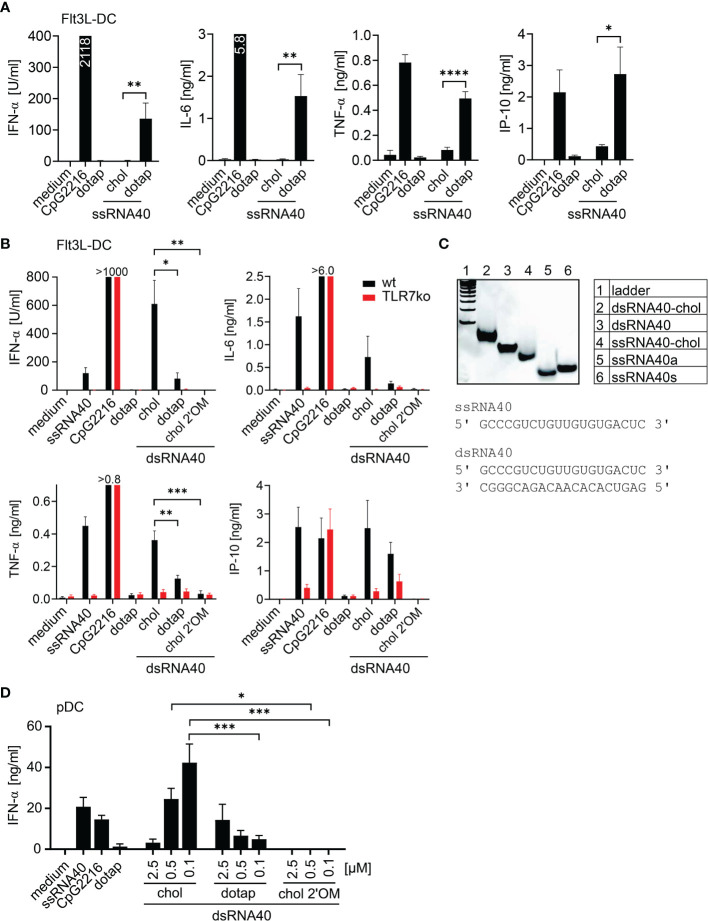
Cholesterol-conjugated dsRNA40 induces a potent immunostimulation. **(A)** Wt Flt3L-DC were stimulated with 0.5 µM ssRNA40-chol or ssRNA40 complex to DOTAP. Medium, CpG2216 (1 µM) and DOTAP served as controls. Supernatants were taken after 20 h of incubation and cytokine production was analyzed by ELISA. Data were calculated from at least five individual experiments. Bars indicate mean + SEM. *p < 0.05, **p < 0.01, ****p < 0.0001; unpaired t-test. **(B)** Wt and TLR7-deficient Flt3L-DC were incubated with 0.5 µM dsRNA40-chol, dsRNA40 complexed to DOTAP and dsRNA40-chol-2**’**OM. Medium ssRNA40 (0.75 µM) complexed to DOTAP, CpG2216 (1 µM) and DOTAP served as controls. Cytokine production was analyzed by ELISA after 20 h of incubation. CpG induced **>**1000 U/ml IFN-α in wt and TLR7-deficient cells, 6.3 ng/ml IL-6 in wt, 6.9 ng/ml IL-6 in TLR7-deficient cells and 0.8 ng/ml TNF-α in wt and 1.0 ng/ml TNF-α in TLR7-deficient cells. Data were calculated from five to seven individual experiments. Bars indicate mean + SEM. *p < 0.05, **p < 0.01, ***p < 0.001; one-way ANOVA with Sidak**’**s *post-hoc* test. **(C)** Polyacrylamide gel electrophoresis of annealed dsRNA40-chol and corresponding sequences. Nucleic acids were stained with SybrGold. One representative gel out of three is shown. **(D)** Purified human pDC were stimulated with dsRNA40-chol, dsRNA40 complexed to DOTAP and dsRNA40-chol-2**’**OM at 2.5 µM, 0.5 µM and 0.1 µM. Medium, ssRNA40 (0.75 µM), CpG2216 (1 µM) and DOTAP served as controls. Supernatants were harvested after 20 h of incubation and IFN-α was analyzed by ELISA. Data were calculated from three independent experiments. Bars indicate mean + SEM. *p < 0.05, ***p < 0.001; one-way ANOVA with Sidak**’**s *post-hoc* test.

In this study, we addressed the immunostimulatory potential of cholesterol-conjugated RNA40. The RNA-sequence used in our study is based on the TLR7 and TLR8 ligand ssRNA40 (1). At first, we analyzed, whether ssRNA40-chol (ssRNA40 with cholesterol-conjugation at the 3**’**-end) could induce cytokine production in murine Flt3L-DC. In contrast to DOTAP-mediated transfection of ssRNA40, ssRNA40-chol did not significantly induce secretion of interferon-α (IFN-α), interleukin-6 (IL-6), tumor necrosis factor-α (TNF-α) or CXCL10 in comparison to DOTAP-mediated transfection of ssRNA40 (**Figure 1A**). Since ssRNA40 could rapidly be digested by cellular RNases, we analyzed the immunostimulatory potential of cholesterol-conjugated dsRNA40, which should be more resistant against degradation by RNases. Indeed, dsRNA40-chol induced significantly higher IFN-α and TNF-α levels compared to dsRNA40 complexed to DOTAP (**Figure 1B**). The cytokines IL-6 and CXCL10 were also induced by dsRNA40-chol (**Figure 1B**). In addition, we wanted to exclude the possibility of cholesterol-induced cytokine release. Because alternating 2**’**O-ribose-methylation (2**’**OM) has been described to render RNA non-stimulatory (19–25) due to the resistance to RNase digestion (7), we stimulated Flt3L-DC with dsRNA40-chol-2**’**OM to silence dsRNA40-mediated immune activation. Accordingly, we could not detect any cytokine-induction arguing against stimulatory activity of cholesterol (**Figure 1B**). Furthermore, dsRNA40-chol-induced cytokine production was strictly dependent on TLR7 since cytokine response was blunted in TLR7-deficient cells (**Figure 1B**). For all experiments on the immunostimulatory potential of dsRNA40-chol, ssRNA40-chol (ssRNA40 with cholesterol-conjugation at the 3**’**-end) and ssRNA40 antisense were annealed at an equal molar ratio and double-strand formation was analyzed by non-denaturing polyacrylamide gel electrophoresis (PAGE) (**Figure 1C**). dsRNA40-chol showed effective annealing and ssRNA40-chol and dsRNA40-chol migrated slower than corresponding unconjugated RNA. Sequences of ssRNA40 and dsRNA40 are also shown in **Figure 1C**.

Due to the fact that dsRNA40-chol leads to TLR7-mediated immunostimulation in the murine system, we purified human pDC and analyzed IFN-α secretion. Indeed, dsRNA40-chol induced IFN-α in human pDC and IFN-α levels were increased with lower concentrations of dsRNA40-chol. In contrast, IFN-α levels induced by dsRNA40 complexed to DOTAP were generally lower and decreased with lower concentrations. This observation supports the conclusion that dsRNA40-chol is superior to dsRNA40 complexed to DOTAP at a concentration of 0.1 µM dsRNA40 (**Figure 1D**). Again, dsRNA40-chol with alternating 2**’**O-ribose-methylation at the antisense strand showed no immunostimulatory activity (**Figure 1D**).

**Figure 2:**
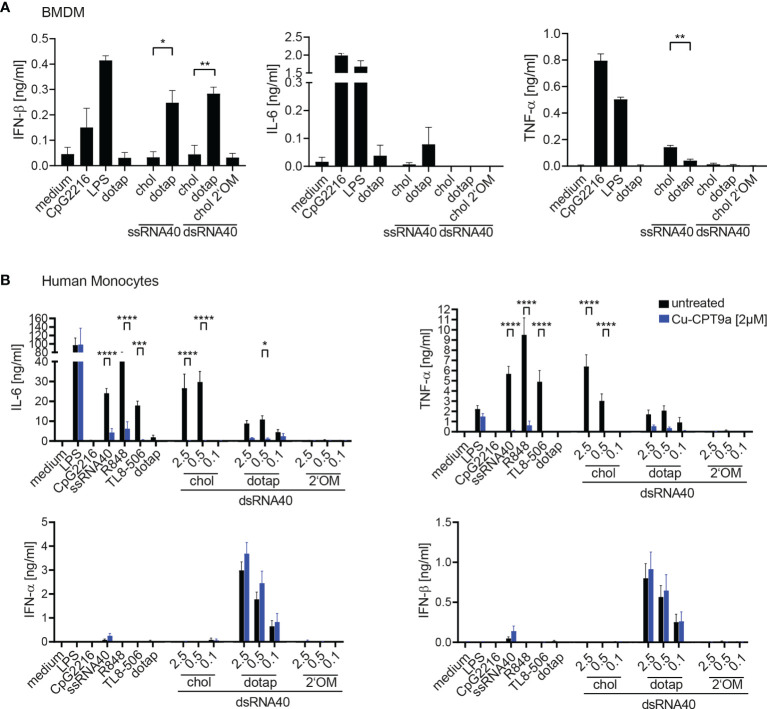
**(A)** Wt BMDMs were stimulated with 0.5 µM ssRNA40-chol or ssRNA40 complex to DOTAP or 0.5 µM dsRNA40-chol, dsRNA40 complexed to DOTAP and dsRNA40-chol-2**’**OM. Medium, CpG2216 (1 µM), LPS (1 µg/ml) and DOTAP served as controls. Supernatants were taken after 20 h of incubation and cytokine production was analyzed by ELISA. Data were calculated from three individual experiments. Bars indicate mean + SEM. Unpaired t-test was used for comparison of ssRNA40 samples while one-way-ANOVA with Sidak’s *post-hoc* test was used for comparison of dsRNA40 samples. *p < 0.05, **p < 0.01, ****p < 0.0001. **(B)** Purified human Monocytes were isolated *via* MACS separation, incubated with (blue bars) or without (black bars) 2 µM of the TLR8 inhibitor Cu-CPT9a and stimulated with dsRNA40-chol, dsRNA40 complexed to DOTAP and dsRNA40-chol-2’OM at 2.5 µM, 0.5 µM and 0.1 µM. Medium, CpG2216 (1 µM), LPS (1 µg/ml), ssRNA/DOTAP(2,5µM), R848 (2,5 µg/ml), TL8-506 (60 nM) and DOTAP served as controls. Supernatants were harvested after 20 h of incubation and IL-6, TNF-α, IFN-α, IFN-β were analyzed by ELISA. Data were calculated from at least three (for TL8-506) to six independent experiments. Bars indicate mean + SEM. *p < 0.05, ***p < 0.001, ****p < 0.0001; two-way ANOVA with Sidak’s *post-hoc* test.

We further extended the analysis of cholesterol-modified RNA40 mediated immune stimulation to murine macrophages and human monocytes (**Figure 2**). The response of murine bone marrow derived macrophages (BMDM) to ssRNA40 was detectable for IFN-β and IL-6 in a DOTAP- dependent manner, whereas only cholesterol-coupled ssRNA40 induced TNF-α (**Figure 2A**). Unexpectedly, dsRNA40-chol did not induce cytokines in BMDM, however DOTAP-complexed dsRNA40 induced IFN-ß, but no IL-6 or TNF-α (**Figure 2A**). In contrast, purified human monocytes responded strongly to dsRNA40-chol with IL-6 and TNF-α production, however type I interferon was not induced (**Figure 2B**). Strikingly, dsRNA40-chol induced cytokine production was strictly TLR8-dependent since the TLR8 inhibitor Cu-CPT9a blunted cytokine production (**Figure 2B**). DOTAP-transfected dsRNA40 also induced IL-6 and TNF-α in a TLR8-dependent manner although at lower levels when compared to dsRNA40-chol. The specificity of the TLR8 inhibitor was verified by demonstrating that cytokine production by stimulation with LPS, a TLR4 ligand, was not influenced by Cu-CPT9a. Of note, IFN-α and IFN-β were only induced by DOTAP-transfected dsRNA40 in a TLR8-independent manner. A response of contaminating pDCs can be ruled out, since the TLR9 ligand CpG2216, a strong IFN-α inducer in pDCs, did not induce any type I interferon in purified monocytes. As a negative control dsRNA40-chol with alternating 2’O-ribose-methylation did not show any immunostimulatory activity (**Figure 2B**).

### Physicochemical Characterization of dsRNA40-Chol

**Figure 3:**
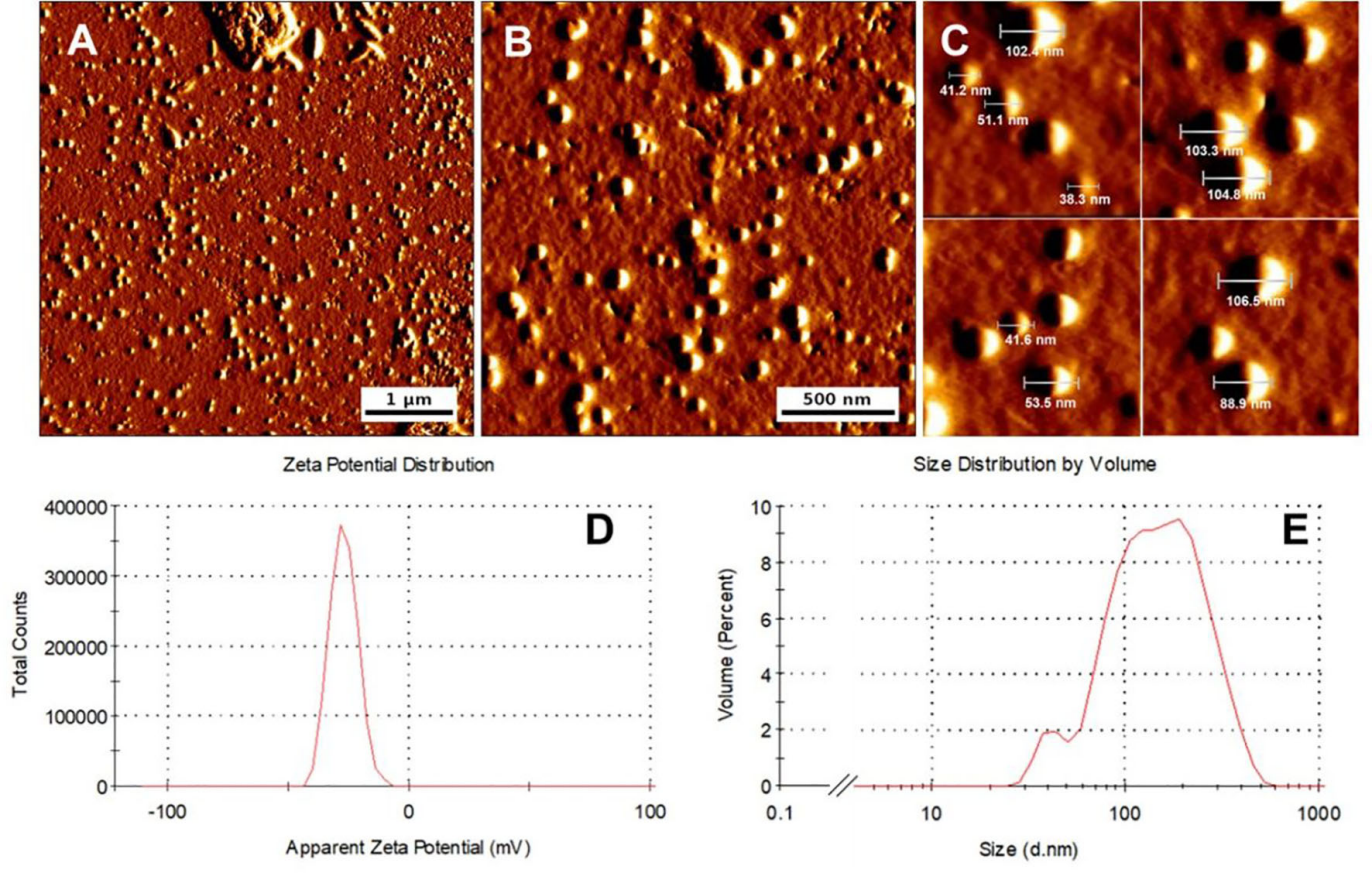
dsRNA40-chol forms nanoparticles by self-assembly. **(A–C)** Morphology of representative dsRNA40-chol nanoparticles shown by atomic force microscopy. All micrographs are presented in amplitude mode. **(D)** represents the LDA zeta potential measurements and **(E)** the size distribution and the diameter of the particles.

Atomic force microscopy (AFM) measurements indicated spherically round shaped dsRNA40-chol nanoparticles formed by self-assembly (**Figures 3A–C**). The LDA analysis of the nanoparticles exhibit a strong negative zeta potential of -29.4 ± 5.5 mV (**Figure 3D**). The particles diameters result from the evaluation of images in the measured height mode. Three particle size fractions are visible by AFM (42.3 ± 4.8 nm about 30%, 81.5 ± 7.4 nm about 65% and a small fraction of 198 ± 11.5 nm) with an average diameter of 79.5 ± 9.4 nm. The prepared particles exhibited hydrodynamic diameters (by PCS) of 41.7 ± 6.4 nm, 122.4± 11.3 nm and 190.1 ± 17.4 nm (distribution by volume, **Figure 3E**) with a mean diameter z-AvE of 97.8 ± 16.5 nm, which are in principle in accordance with the AFM measurements. It should however be noted that the differences in size arising from the PCS and AFM measurements is because the hydrodynamic diameter obtained in aqueous conditions while the latter is measured at atmospheric conditions.

### dsRNA40-Chol Nanoparticles Are Efficiently Taken Up by Different Immune Cells

**Figure 4:**
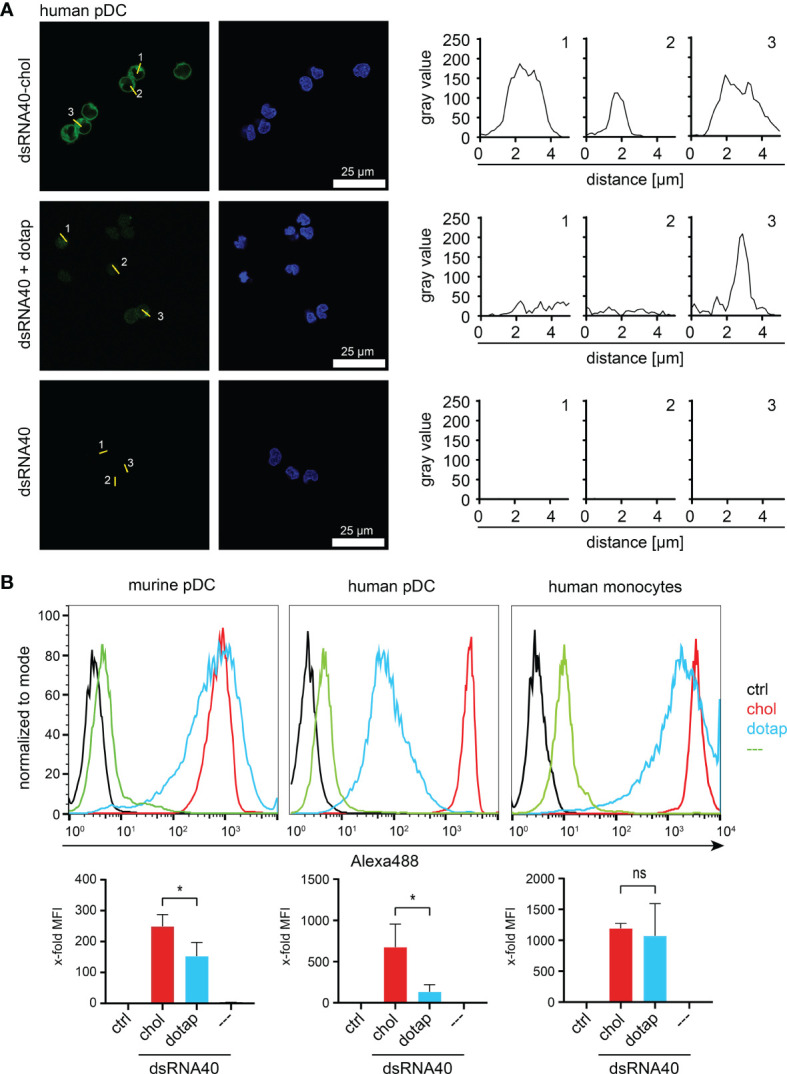
dsRNA40-chol nanoparticles are efficiently taken up by different immune cells. **(A)** Purified human pDC were incubated with 0.5 µM of Alexa488-labeled dsRNA40-chol, dsRNA40 complexed to DOTAP or dsRNA40. Cells were extensively washed and fixed after 1.5 h of incubation and analyzed by confocal laser scanning microscopy. RNA40 is shown in green and DAPI-stained nuclei in blue (left panel, scale bar 25 µm). In the right panel, the fluorescence intensity profile is plotted as grayscale (fluorescence intensity) versus distance (in µM) for individual cells. For every cell stimulation condition, 3 cells were marked by a line (numbered from 1 to 3) and corresponding fluorescence profile (numbered from 1 to 3) plotted along this line. **(B)** Murine Flt3L-DC (B220+/CD11c+ pDC), purified human pDC and monocytes were incubated as described in **(B)** and uptake of RNA40 was analyzed by flow cytometry. Histograms depict one representative experiment out of three independent experiments. Bar charts combine median fluorescent intensities (MFI) of three independent experiments. Values were normalized to ctrl. Bars indicate mean + SEM. ns: not significant, *p < 0.05; one-way ANOVA with Sidak’s post-hoc test.

To address the uptake of self-assembled dsRNA40-chol nanoparticles by immune cells, we incubated human and murine pDC and human monocytes with Alexa488-labeled dsRNA40-chol, Alexa488-labeled dsRNA40 complexed to DOTAP or Alexa488-labeled dsRNA40 without any conjugation or complex formation. Cells were analyzed by confocal laser scanning microscopy and FACS. Importantly, dsRNA40-chol was taken up efficiently by human pDC when compared to naked dsRNA40 or RNA40 complexed to DOTAP (**Figure 4A**). Similarly, quantification of RNA uptake by murine and human pDCs as well as human monocytes using flow cytometry showed significantly improved uptake of dsRNA40-chol compared to naked or complexed dsRNA40 (**Figure 4B**).

### dsRNA40-Chol Nanoparticles Induce Proinflammatory Cytokines in Purified Human Monocytes and Promote T_H1_ and T_FH_ Cell Responses

**Figure 5:**
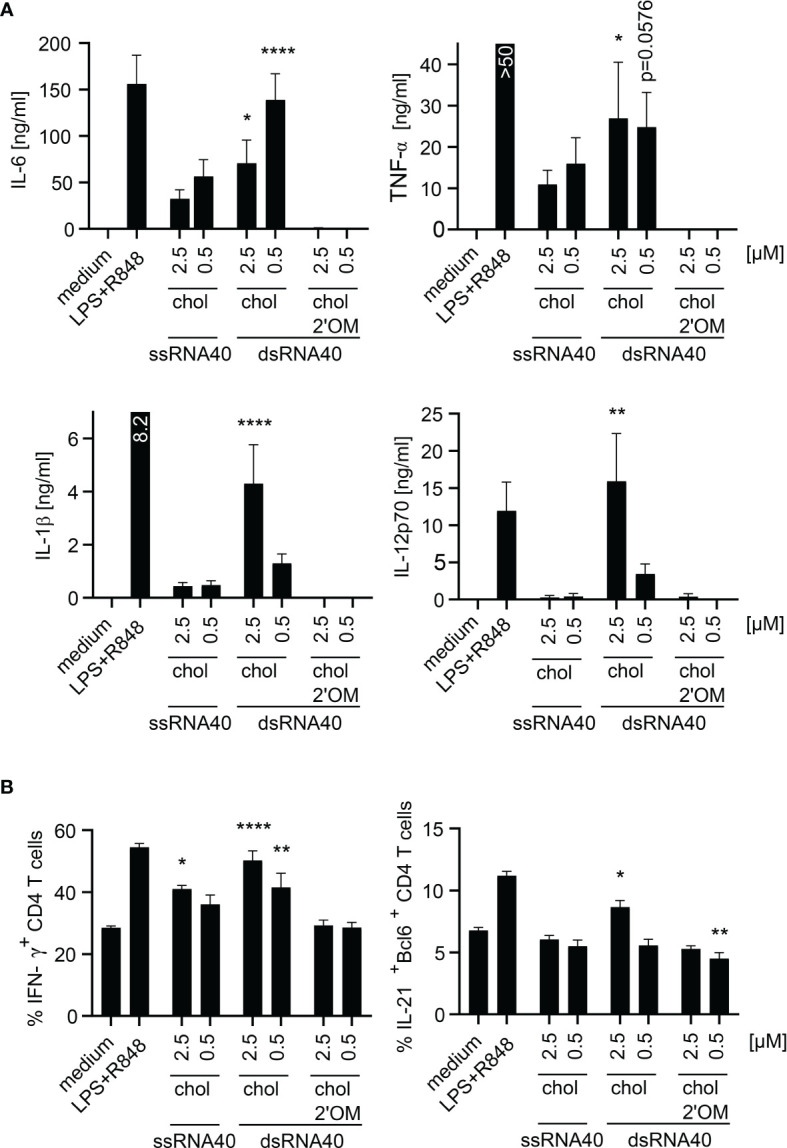
dsRNA40-chol nanoparticles induce proinflammatory cytokines in purified human monocytes and promotes T_H1_ and T_FH_ cell responses. **(A)** Purified human monocytes were stimulated with ssRNA40-chol, dsRNA40-chol and dsRNA40-chol-2’OM at 2.5 µM and 0.5 µM. Medium and LPS (1 µg/ml) in combination with R848 (2.5 µg/ml) served as controls. Supernatants were harvested after 20 h of incubation and cytokine induction was analyzed by ELISA. Data were calculated from six individual donors. Bars indicate mean + SEM. *p < 0.05, **p < 0.01, ****p < 0.0001; one-way ANOVA with Dunnett’s *post-hoc* test. **(B)** Purified human CD4^+^ T cells were restimulated with anti-CD3 and anti-CD28 and incubated with conditioned monocyte supernatant [monocytes were stimulated as described in **(A)**]. Cells were incubated for five days before they were analyzed by flow cytometry. Data were calculated from four individual donors. Bars indicate mean + SEM. *p < 0.05, **p < 0.01, ****p < 0.0001; one-way ANOVA with Dunnett’s *post-hoc* test.

To study the adjuvant potential of ssRNA40-chol or dsRNA40-chol nanoparticles we set up an *in vitro* T-cell differentiation assay where CD3- and CD28-activated CD4^+^ T cells were incubated with supernatant from stimulated monocytes containing differentiation-promoting cytokines (26). We focused in particular on the differentiation of IFN-γ-producing T helper type 1 cells (T_H1_) and IL-21-producing T follicular helper cells (T_FH_). T_H1_ cell play a fundamental role in the host defense response against intracellular pathogens, whereas T_FH_ cells are important regulators of the germinal-center response and humoral immunity by interacting with B-cells (27). Accordingly, we stimulated purified human monocytes with ssRNA-chol or dsRNA-chol and used the supernatants for T cell differentiation. The analysis of the supernatants showed that dsRNA40-chol induced IL-6, TNF-α, IL-1β and IL-12p70. IL-6 production was higher at 0.5 µM of dsRNA40-chol in comparison to 2.5 µM, whereas TNF-α, IL-1β and IL-12p70 secretion was more prominent at 2.5 µM (**Figure 4A**). Interestingly, ssRNA40-chol showed some cytokine (e.g. IL-6 and TNF-α) induction in human monocytes (**Figure 5A**) although less potent than dsRNA40-chol. Again, we could not detect any cytokine production induced by dsRNA40-chol with alternating 2’O-ribose-methylation at the antisense strand (**Figure 5A**). To determine the contribution of RNA40-chol induced innate immunostimulation to human T cell differentiation, we analyzed whether supernatants harvested from RNA40-chol stimulated monocytes had any influence on T cells, in particular the differentiation of IFN-γ-producing T_H1_ and IL-21-producing T_FH_ cells. Indeed, CD4^+^ T cells showed significantly increased intracellular IFN-γ expression if activated by CD3 and CD28 in presence of supernatants of purified monocytes stimulated with 2.5 µM of ssRNA40-chol and 2.5 µM or 0.5 µM of dsRNA40-chol (**Figure 5B**). Additionally, monocytes stimulated with 2.5 µM of dsRNA40-chol were able to induce expression of the transcription factor Bcl-6, which is important for T_FH_ cell development (28, 29) and IL-21 in CD4^+^ T cells (**Figure 5B**). In summary, ssRNA40-chol promotes T_H1_ cell differentiation, whereas dsRNA40-chol promotes T_H1_ as well as T_FH_ cell differentiation.

## Discussion

Immunostimulation of the innate immune system with subsequent activation and tuning of the adaptive immune response relies on activation of germline-encoded PRR by pathogen-associated molecular patterns (PAMP). Amongst other PRR, ssRNA as well as dsRNA have been reported to stimulate the immune system *via* TLR7 and TLR8 (1, 2). More precisely, both TLR recognize RNA degradation products present as nucleosides and oligoribonucleotides. TLR7 detects guanosine and uridine moieties in ssRNA (4) and TLR8 recognizes uridine and in addition a short oligonucleotide created by RNase T2 and/or RNase 2 (5–7). Cellular uptake of nucleic acids into the endosomal compartment, where TLR that recognize nucleic acids are localized (30, 31), has to be facilitated to analyze nucleic acid-induced immune responses. Cells are usually transfected with liposome-nucleic acid complex consisting of nucleic acid molecules complexed to transfection reagents like DOTAP, because otherwise nucleic acids hardly interact with negatively charged cell membranes and are highly prone to degradation by RNases or DNases before they reach the endosomal compartment. Common transfection strategies require an initial liposome-nucleic acid complex formation step prior to adding the immune activator/TLRL to target cells, which then enters the cells *via* endocytosis-like processes (32, 33) or by temperature-independent transport mechanisms through cell membranes (33).

Therefore, we focused on characterization of a simple system that does not depend on the requirement of initial liposome-nucleic acid complex formation to activate the immune system by the TLR7 and TLR8 ligand RNA40. We analyzed the immunostimulation in plasmacytoid dendritic cells induced by ssRNA40-chol containing a phosphodiester backbone but could not detect significant amounts of IFN-α, IL-6, TNF-α and CXCL10 in comparison to ssRNA40 complexed to DOTAP. This outcome was most likely due to increased susceptibility of ssRNA40-chol to RNAse-digestion. Forsbach et al. (11) have shown that cholesterol-conjugated ssORN with highly nuclease resistant phosphorothioate nucleotide linkage activates human PBMC and murine spleen DC to produce IFN-α and TNF-α. Furthermore, they described that mice responded with CXCL10 secretion after i.v. injection of ssORN-chol containing a phosphorothioate backbone (11). However, dsRNA conjugated to cholesterol was not tested. In addition, it has been described for antisense oligonucleotide delivery that it may be important to cleave the phosphodiester between cholesterol and antisense oligonucleotide intracellularly (25, 26). For that reason, cholesterol-conjugation with phosphorothioate RNA40 may be less active for recognition by PRR. Additionally, toxicities in association with phosphorothioate linkage have been reported (27). Furthermore, we hypothesized that RNase-digest of dsRNA40-chol leads to higher amounts of TLR7/TLR8 stimulatory fragments in contrast to ssRNA40-chol which could be digested in total. Therefore, we investigated the immunostimulatory potential of dsRNA40-chol instead and detected that dsRNA40-chol was superior to dsRNA40 complexed to DOTAP in its ability to induce IFN-α and TNF-α in murine Flt3L-DC. IL-6 and CXCL10 secretion was also induced by dsRNA40-chol and cytokine induction was strictly dependent on TLR7 in murine Flt3L-DC. In contrast, human monocytes responded strongly with IL-6 and TNF-α production to dsRNA40-chol stimulation in a strictly TLR8-dependent manner. Since TLR8 in mice is non-responsive to RNA ligands (17, 34, 35), murine bone marrow derived macrophages (BMDM) were not stimulated by dsRNA40-chol. In contrast, DOTAP-complexed dsRNA40 induced strong type I interferon production in a TLR8-independent fashion suggesting that TLR7 in monocytes is activated by this formulation. Our results contrast a recent study by de Marcken M et al. (36) that describes type I interferon production by TLR8 and not TLR7 in human monocytes. The reason for this discrepancy is currently unknown and may be dependent on the different systems used.

We further characterized the dsRNA-chol formulation by atomic force microscopy and photon correlation spectroscopy. This analysis showed, that dsRNA40-chol forms nanoparticles of three particle size fractions. Currently, we do not know why dsRNA40-chol generates heterogeneous nanoparticles, but for further advancing this formulation to a clinical application, it would be necessary to control particle size and formation. In addition, this self-assembly of dsRNA40-chol nanoparticles is somewhat reminiscent of the formation of immune stimulating complexes (ISCOMs) that are spherical cage-like nanoparticles consisting of the saponin adjuvant Quil A, cholesterol, phospholipids and antigens (37). However, the dsRNA40-chol nanoparticles are devoid of antigen and future experiments should focus on integrating antigen into these nanoparticles. Thorough analysis of the dsRNA-chol nanoparticles demonstrated that they are taken up by murine pDC, human pDC and human monocytes and are efficiently internalized. Within the human system, we found that dsRNA40-chol lead to secretion of IFN-α in purified pDC. Purified human monocytes responded with IL-6, TNF-α, IL-1β and IL-12p70 secretion. Comparison of murine Flt3L-DC/human pDC with human monocytes showed that higher amounts of dsRNA40-chol nanoparticles are needed to activate monocytes. These observations are confirmed by studies by Rettig et al. (38). They found that human pDC are efficient in taking up immunostimulatory protamine-ssRNA nanoparticles and respond with IFN-α secretion. Furthermore, they could show that human monocytes are able to internalize nano- as well as microparticles and exhibit a higher threshold of activation in contrast to pDC. Interestingly, human monocytes produced IL-6 and TNF-α in response to ssRNA40-chol as well. This could be due to higher phagocytotic activity of monocytes in comparison to pDC. Our finding that dsRNA40-chol induces IL-1β in human monocytes indicates that dsRNA40-chol cannot only activate murine TLR7 but also activates human TLR8. This is supported by investigations of human monocytes showing that IL-1β secretion after HIV infection depends mainly on TLR8 and only to minimal extent on TLR7 (39) and by data demonstrating that TLR8, and not TLR7, activates the NLRP3 inflammasome resulting in IL-1β secretion (40). This observation is further supported by the fact that TLR8 is in contrast to TLR7 highly expressed in human monocytes (26, 41). Another aspect, which supports TLR8 activation by RNA40-chol is that TLR8 and not TLR7 activation has been demonstrated to promote T_H1_ cells (36), which is in line with our data showing that dsRNA40-chol and ssRNA40-chol drove T_H1_ cell differentiation. In addition, dsRNA40-chol induced T_FH_ cell differentiation, which depends like T_H1_ cell differentiation on IL-12p70 (26, 42, 43) and secretion of IL-12p70 by human monocytes depends on TLR8 and is non-redundant with other TLR (44, 45). Of note, it has just recently been reported that activation of TLR8 by dsRNA40 requires RNase 2 as well as RNase T2 and both RNases cooperate to cleave dsRNA substrates (7).

To exclude an immunostimulatory potential by cholesterol, we used dsRNA40-chol with alternating 2’O-ribose-methylation, which renders immune activating RNA non-stimulatory (19–25) because it is neither digested by RNase 2 nor RNase T2 (7), and found no stimulatory effect in all cell types tested. Additionally, *in vivo* studies showed that chol-conjugated nucleic acids are taken up into several tissues (9, 10, 46). The most important one is the liver since high-density lipoprotein (HDL) and especially low-density lipoprotein (LDL) are important delivery proteins for cholesterol-conjugated nucleic acids (9, 10). Given that observation in addition to the knowledge that IFN-α induction by TLR7 agonists is of high interest during chronic hepatitis B virus infection (47–49), conjugation of TLR7-ligands to cholesterol could be a therapeutical approach. Since cholesterol-conjugated nucleic acids are predominantly delivered to liver, kidney and spleen (46), all belonging to primary clearance tissues, these organs are potential targets for dsRNA40-chol as TLR7/8 agonist.

In summary, we investigated the immunostimulatory potential of cholesterol-conjugated dsRNA40 and demonstrated efficient internalization and cytokine induction in various immune cells with the potential of driving T cell differentiation. Therefore, dsRNA40-chol may be a simple and potent adjuvant for future clinical use without the need for separate carriers such as liposomes.

## Data Availability Statement

The raw data supporting the conclusions of this article will be made available by the authors, without undue reservation.

## Ethics Statement

The local ethics committees of Justus-Liebig-University Giessen and Philipps-University Marburg reviewed and approved the use of human blood samples for this study and written informed consent was obtained from the blood donors. For experiments with murine immune cells, mice were sacrificed and tissue/organs removed. These experiments were performed in accordance with the National German welfare law §4 (3) TierSchG and §2 and Annex 2 (TierSchVerV) of the National Order for the use of animals in research and do not need the approval by a local ethics committee. According to the regulations, the number of mice used was reported to the animal welfare officer of the Philipps-University Marburg.

## Author Contributions

H-LO, IL, JS, JD, KE, and UB performed experiments. H-LO, LES, UB, AK, and SB designed the experiments. H-LO, AK, JS, LES, UB, and SB analyzed the data. H-LO and SB wrote the manuscript. H-LO and SB conceived and supervised the study. All authors contributed to the article and approved the submitted version.

## Funding

This work was funded by the German Center for Infection Research (TTU 09.806 to SB); the Deutsche Forschungsgemeinschaft (DFG, German Research Foundation) Project-ID 369799452 TRR237 - A02 to SB and Project-ID 114933180 TR84 - C10 to SB and LES.

## Conflict of Interest

The authors declare that the research was conducted in the absence of any commercial or financial relationships that could be construed as a potential conflict of interest.

## Publisher’s Note

All claims expressed in this article are solely those of the authors and do not necessarily represent those of their affiliated organizations, or those of the publisher, the editors and the reviewers. Any product that may be evaluated in this article, or claim that may be made by its manufacturer, is not guaranteed or endorsed by the publisher.
